# Creation and Worldwide Utilisation of New COVID-19 Online Information Hub for Genetics Health Professionals, Patients and Families

**DOI:** 10.3389/fgene.2021.621683

**Published:** 2021-07-08

**Authors:** Adam P. Tobias, Jonathan Berg, Roseanne Cetnarskyj, Zosia Miedzybrodzka, Mary E. Porteous, Edward S. Tobias

**Affiliations:** ^1^Edinburgh Medical School, College of Medicine and Veterinary Medicine, University of Edinburgh, Edinburgh, United Kingdom; ^2^Department of Clinical Genetics, Ninewells Hospital and Medical School, University of Dundee, Dundee, United Kingdom; ^3^Scottish Genetics Education Network, University of Dundee, Kirkcaldy, United Kingdom; ^4^School of Health Sciences, University of Dundee, Kirkcaldy, United Kingdom; ^5^Medical Genetics Group, University of Aberdeen, Aberdeen, United Kingdom; ^6^South East Scotland Genetic Service, Western General Hospital, Edinburgh, United Kingdom; ^7^Academic Unit of Medical Genetics and Clinical Pathology, Queen Elizabeth University Hospital, University of Glasgow, Glasgow, United Kingdom; ^8^School of Medicine, Dentistry and Nursing, College of Medical, Veterinary and Life Sciences, University of Glasgow, Glasgow, United Kingdom; ^9^West of Scotland Centre for Genomic Medicine, Queen Elizabeth University Hospital, NHS Greater Glasgow and Clyde, Glasgow, United Kingdom

**Keywords:** COVID–19, online, genetics, education, coronavirus, genomics, resources, data

## Abstract

The current COVID-19 pandemic has unfortunately resulted in many significant concerns for individuals with genetic disorders and their relatives, regarding the viral infection and, particularly, its specific implications and additional advisable precautions for individuals affected by genetic disorders. To address this, the resulting requirement for guidance and information for the public and for genetics professionals was discussed among colleagues nationally, on the ScotGEN Steering Committee, and internationally on the Education Committee of the European Society of Human Genetics (ESHG). It was agreed that the creation of an online hub of genetics-related COVID-19 information resources would be particularly helpful. The proposed content, divided into a web page for professionals and a page for patients, was discussed with, and approved by, genetics professionals. The hub was created and provided online at www.scotgen.org.uk and linked from the ESHG’s educational website for genetics and genomics, at www.eurogems.org. The new hub provides links, summary information and representative illustrations for a wide range of selected international resources. The resources for professionals include: COVID-19 research related hubs provided by Nature, Science, Frontiers, and PubMed; clinical guidelines; the European Centre for Disease Prevention and Control; the World Health Organisation; and molecular data sources including coronavirus 3D protein structures. The resources for patients and families include links to many accessible sources of support and relevant information. Since the launch of the pages, the website has received visits from over 50 countries worldwide. Several genetics consultants have commented on usefulness, clarity, readability, and ease of navigation. Visits have originated most frequently in the United Kingdom, Kuwait, Hong Kong, Moldova, United States, Philippines, France, and Qatar. More links have been added since the launch of the hub to include additional international public health and academic resources. In conclusion, an up-to-date online hub has been created and made freely available for healthcare professionals, patients, relatives and the public, providing categorised easily navigated links to a range of worldwide resources related to COVID-19. These pages are receiving a rapidly growing number of return visits and the authors continue to maintain and update the pages’ content, incorporating new developments in this field of enormous worldwide importance.

## Introduction

In December 2019, it was recognised that the severe acute respiratory syndrome coronavirus 2 (SARS-CoV-2), was being transmitted through the human population and causing the potentially fatal human coronavirus disease, COVID-19. During the ensuing global pandemic, there has been enormous internet search activity worldwide for online information in relation to the virus and the disease, especially at the times of major announcements made by the World Health Organisation (WHO) (Szmuda et al., 2020a). The response from the scientific community to the COVID-19 pandemic has been rapid and extensive. Many different aspects of the disease have already been investigated and continue to be explored. As a consequence, numerous articles have been written, published, and made widely available (Cevik et al., 2020).

Many highly informative online resources in relation to COVID-19 and to the causative coronavirus have now been created. These include online hubs directly linking to original genetic scientific publications, such as the Frontiers Coronavirus Knowledge Hub in addition to those provided by Nature Journals, Science Journal, Pubmed LitCovid, the RCSB (Research Collaboratory for Structural Bioinformatics) Protein Data Bank and institutions such as the National University of Singapore. Other websites have been established to provide more summarised information for professionals, such as Nextstrain (with its animated representations of sequence-based coronavirus global epidemiological data) and also the European Reference Networks. Many of these COVID-19 information resources have been relatively recently created and may not all be well known or easily located online amongst other websites.

Although many COVID-19-related resources are accessible by the public it has been reported that many of the information sources are not easily accessible by that audience (Szmuda et al., 2020b). There are, however, several excellent COVID-19-related resources that have been created specifically for the public and, in particular, for patients affected by genetic disorders. These include the COVID-19 resources provided by Unique, the Genetic Alliance and the Contact organisation. Relevant and helpful online resources have also been created by national specialist organisations and major institutions such as the British Society for Genetic Medicine, US Centers for Disease Control and Prevention (CDC), UK National Health Service, European Centre for Disease Prevention and Control, New York’s Mount Sinai Icahn School of Medicine, University of Hong Kong, Clinic Barcelona University Hospital and the World Health Organisation.

The Scottish Genetic Education Network (ScotGEN) was created by the four Scottish centres for Clinical Genetics, collaboratively with Scottish Universities, and was launched in 2005. It links all of those individuals who teach genetics for healthcare in Scotland and its website (www.ScotGEN.org.uk) provides a shared national online hub for relevant learning and teaching resources (Scottish Genetic Education Network, 2021). These encompass a wide range of genetics and genomics educational topics, including educational genomics apps (Tobias and Tobias, 2015). The website thus provides extensive information, in addition to many carefully selected web-links, for professionals and patients, to facilitate understanding of genetics and its application to everyday practice (Scottish Genetic Education Network, 2021).

### Objectives

In view of the quantity and quality of individual COVID-19-related resources that had become established online around the world that were of great interest and usefulness to clinical and non-clinical genetics professionals and to the public, it was perceived by the authors, including educators and genetics health professionals, that it would be highly beneficial to create a freely accessible centralised online hub, providing direct links to a range of free, high-quality, informative and up-to-date websites. Also, given the large range of information sources available online, including some that may be less suitable for the public (Szmuda et al., 2020b), the authors have created an accompanying guide to a range of those online sources that have been generated for the public (including affected individuals and their family members).

The authors have, in this way, created an online hub providing (a) a page of concisely described links aimed at professionals, including links to websites providing regularly updated highlights (and comprehensive searches) of peer-reviewed original scientific research articles and (b) a page of similarly annotated links for patients and their families. These were added, prominently, to the existing educational web pages of the Scottish Genetic Education Network at www.ScotGEN.org.uk (Scottish Genetic Education Network, 2021).

## Materials and Methods

### Initial Selection of Linked Sources

In selecting online resources to be linked directly from the new ScotGEN COVID-19 pages, a method was used that was similar to that which had been previously used in creating the EuroGEMS.org website (Tobias and Tobias, 2020). Approximately 35 websites were initially considered. The web resources were initially identified by: personal web-searches; personal use of the resources; suggestions from professional colleagues; checking the websites of major organisations in the field of genetics designed for professionals and also for patients; consulting other resources often used as reference sources by major international organisations; and the COVID-19-specific literature hubs run by major scientific publishers. Decisions with regard to source inclusion were made by this article’s lead and corresponding author, based on the following inclusion criteria for online resources: (a) free-to-access, (b) high-quality (containing information that was judged as being reliable and free of obviously misleading information), (c) containing up-to-date links, without broken URLs (or “404 errors”) that would suggest a failure to maintain and update the online resource, (d) useful (judged as being likely to be helpful to and understandable by the viewer), and (e) informative (providing relevant information and details). Fulfilment of all criteria was regarded as essential for inclusion. The criteria and content were discussed in detail with other professional colleagues.

In order to facilitate navigation by a user, the sources were grouped, as planned, into a page of COVID-19 Resources for genetics professionals and a separate page for patients and families affected by genetic disorders. In addition, to aid online navigation, for each linked online source, a short summary of its content and representative image was included, in addition to its title.

The new web pages and their content were discussed with a large group of clinical genetics colleagues in the West of Scotland Centre for Genomic Medicine, which serves a population of approximately 3 million. These colleagues included clinical genetic counsellors and consultants who each have many years of experience in discussing and explaining scientific concepts to members of the public, including patients and their relatives, verbally and also in printed and electronic form. The new web pages were also shown to two family doctors (general practitioners) and five individuals who were not healthcare professionals. The pages were also discussed with the members of the ScotGEN Steering Committee and with the Education Committee of the European Society of Human Genetics. Where appropriate, any suggested additional COVID-19-related websites meeting the selection criteria were added if they had not already been incorporated.

### Technical Aspects of Web-Page Creation

The webpages were created using a “waterfall” methodology, which involves the step-by-step completion of a linear (non-circular) sequence of stages and is a method often used in the development of educational websites and software. The first stage of development was research into different sites and familiarisation with the development stack which was already being used for the website. Two new pages (a professional resource list and a patient resource list) would be added to the website, as well as a modification to the homepage as an internal link. Low fidelity wireframes were then created, using some elements of the User Interface (UI) which already existed in other pages of the site in order to maintain consistency. First prototypes of the webpages were then implemented using the Bootstrap CSS library, as this was the language used for front-end development on the remainder of the website, maintaining the appearance of the website as well as maximising the loading efficiency of the webpages. Mobile optimisation was incorporated by hiding the image on each row which allows for larger text display if the screen is smaller. Web page speed was analysed using Google PageSpeed Insights (Google PageSpeed Insights, 2021).

In order to increase accessibility to potential users, additional links to the website were placed on the appropriate web-pages of the existing educational genetics web pages run on behalf of the European Society of Human Genetics at www.EuroGEMS.org (ESHG Genetic Educational Materials and Sources, 2021). Thus, a prominent link was placed on that website’s page for genetics professionals and also on the page for patients and families affected by genetic conditions.

In order to be able to monitor visitor numbers to the new pages, a General Data Protection Regulation (GDPR)-compliant online service (Statcounter) was used (Statcounter, 2021).

## Results

The new pages were created as planned, with the page for genetics professionals containing a variety of informative resources (Table 1). These included sources relating to (a) COVID-19-related practical advice for healthcare, (b) Nature, Science, Frontiers, LitCovid (PubMed), and RCSB Protein Data Bank (PDB) hubs for Coronavirus research publications, (c) the COVID-19 pages of international organisations such as the World Health Organisation, European Centre for Disease Prevention and Control, and European Reference Networks, and (d) Nextstrain. Each of these websites contain much relevant information. For example, the RCSB PDB now contains data for a large number of structures for coronavirus molecules, including 3D molecular data delineating the structure of the coronavirus spike protein bound to ACE2 or to antibodies. The PDB data are freely available and are linked to the relevant research publications.

**TABLE 1:** Website names and URLs for all of the websites to which links are provided in the new web-page entitled “COVID-19 Resources for Healthcare Professionals” on the www.ScotGEN.org.uk website.

**Website**	**Current URL**
European Reference Networks (ERNs) and patient organisations	http://international.orphanews.org/summary/editorial/nl/id-200327.html
General Practitioner / GP Notebook: COVID-19 resources	https://gpnotebook.com/covid19.cfm
NHS Education for Scotland: COVID-19 Learning Materials	https://learn.nes.nhs.scot/27993/coronavirus-covid-19
European Centre for Disease Prevention and Control	https://www.ecdc.europa.eu/en
World Health Organisation	https://www.who.int
Nature Journals Coronavirus hub	https://www.springernature.com/gp/researchers/campaigns/coronavirus
Science Journal Coronavirus hub	https://www.sciencemag.org/collections/coronavirus?IntCmp=coronavirussiderail-128
LitCovid (NIH curated Coronavirus literature hub)	https://www.ncbi.nlm.nih.gov/research/coronavirus/
The Frontiers Coronavirus Knowledge Hub	https://coronavirus.frontiersin.org/?utm_campaign=sub-cov-cco&utm_medium=fhpc&utm_source=fweb
The National University of Singapore (School of Public Health) COVID-19 Research hub	https://sph.nus.edu.sg/covid-19/research/
Coronavirus—The Science Explained—An overview from the UKRI	https://coronavirusexplained.ukri.org/en/
Horizon	https://horizon-magazine.eu/topics/health
Nextstrain	https://nextstrain.org
RCSB Protein Data Bank	http://www.rcsb.org

In a similar way, the new web page for patients and their families contains descriptions of, and direct links to, several relevant organisations, such as Unique, Genetic Alliance, WellChild and Contact, providing advice regarding COVID-19 for individuals with rare genetic disorders, that includes practical guidance, resources and sources of assistance. The relevant resources for the public, to which links are provided, also include those provided by the WHO, the European Centre for Disease Prevention and Control and the US Centers for Disease Control and Prevention (CDC), in addition to the Fight-COVID-19 website of Hong Kong University and the Facts and Resources web pages of Mount Sinai Medical School in New York. The full list of included websites is outlined in Table 2. The authors would welcome recommendations for additional links.

**TABLE 2:** Website names and URLs for all of the websites to which links are provided in the new web-page entitled “COVID-19 Resources for Patients and Families” on the ScotGEN.org.uk website.

**Website**	**Current URL**
Coronavirus—The Science Explained	https://coronavirusexplained.ukri.org/en/
Unique Website	https://www.rarechromo.org/covid19update/
WellChild Website	https://www.wellchild.org.uk/2020/03/18/ten-ways-to-keep-my-child-with-complex-health-needs-safe/
Genetic Alliance & Rare Disease UK pages	https://geneticalliance.org.uk/news-events/
The “Contact” Organisation	https://www.contact.org.uk/advice-and-support/coronavirus-information-for-families-with-disabled-children/
European Centre for Disease Prevention and Control	https://www.ecdc.europa.eu/en/COVID-19/national-sources
UK NHS Guidelines	https://www.nhs.uk/conditions/coronavirus-covid-19/
US Guidelines (CDC)	https://www.cdc.gov/coronavirus/2019-ncov/index.html
Fight COVID-19 (Hong Kong University)	https://fightcovid19.hku.hk
Facts and Resources (Mount Sinai, New York)	https://www.mountsinai.org/about/covid19
Clinic Barcelona (University Hospital): COVID-19	https://www.clinicbarcelona.org/en/assistance/diseases/covid-19
World Health Organisation	https://www.who.int
Detailed review of the origin of COVID-19	https://www.ncbi.nlm.nih.gov/pmc/articles/PMC7995093/
NHS UK: Coronavirus vaccine	https://www.nhs.uk/conditions/coronavirus-covid-19/coronavirus-vaccination/coronavirus-vaccine/
NHS Inform. The coronavirus vaccine. Side effects.	https://www.nhsinform.scot/covid-19-vaccine/the-vaccines/side-effects-of-the-coronavirus-vaccines
Oxford University Hospitals: COVID-19 FAQs	https://www.ouh.nhs.uk/working-for-us/staff/covid-staff-faqs-vaccine.aspx

### International Use of the ScotGEN COVID-19 Web Pages

The new COVID-19 pages on ScotGEN’s website have already attracted many visitors from around the world. The visitors to the website include countries in North and South America (e.g., Brazil), Africa (e.g., Algeria), Europe, Asia (e.g., Kuwait, Japan and Singapore), and New Zealand.

**FIGURE 1:**
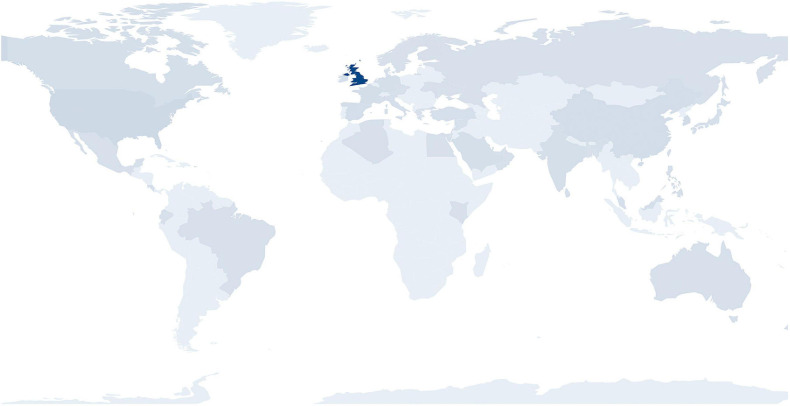
Countries and regions of origin of visitors to the new COVID-19 web pages in the first year since launch. The intensity of shading relates to the proportion of total visits.

The web pages were made publicly available on 24th April 2020. Using data from Statcounter.com (Statcounter, 2021), since launch of the COVID-19 webpages (approximately 1 year ago) the website has received approximately 7175 page views (or 19–20 per day, on average), including those visits (representing approximately 64% of the total) that could not be recorded as a result of the reported rejection or blocking of cookies by internet browsers and users (Sullivan, 2020). Of the visits to the new pages that could be recorded, 53.8% of visits were to the page for patients and families, with the remaining 46.2% to the page for professionals. The websites’ visitors have originated in over 50 countries (see Figure 1 and Table 3) and the frequency of “returning visits” has increased by over fivefold, over a 6-month period (Statcounter, 2021).

**TABLE 3:** Countries or region of origin and respective proportions of website visits, in the first year since launch of the COVID-19 webpages.

**Country or region**	**% of page views**
United Kingdom	78.9
United States	3.5
Korea, Republic of	2.2
China	1.5
Kuwait	1.4
France	1.3
Spain	1.1
Saudi Arabia	0.8
Hong Kong	0.7
Germany	0.7
India	0.6
Canada	0.6
Philippines	0.5
Netherlands	0.4
Finland	0.4
Denmark	0.4
Malaysia	0.4
Turkey	0.3
Moldova, Republic of	0.3
Italy	0.3
Singapore	0.3
Qatar	0.3
Japan	0.3
New Zealand	0.2
Ireland	0.2
Costa Rica	0.2
Sweden	0.1
Oman	0.1
Israel	0.1
Egypt	0.1
Algeria	0.1
Czech Republic	0.1
Brazil	0.1
Bangladesh	0.1
Australia	0.1
Austria	0.1
Ukraine	0.1
Taiwan	0.1
Syrian Arab Republic	0.1
Russian Federation	0.1
Puerto Rico	0.1
Norway	0.1
Mexico	0.1
Malta	0.1
Luxembourg	0.1
Sri Lanka	0.1
Kenya	0.1
Greece	0.1
Europe	0.1
Estonia	0.1
Ecuador	0.1
Bahrain	0.1
United Arab Emirates	0.1

The websites most frequently visited from the new pages are those for Nature Journals Coronavirus Research (from the professionals’ page) and UKRI Coronavirus Explained (from the page for patients and their families) (Table 4).

**TABLE 4:** Names and URLs of the 10 most frequently used exit links from the new COVID-19 ScotGEN pages, together with the respective proportions of the total visits to external websites.

**Website visited**	**% of total**	**Exit link (URL used)**
Nature—Coronavirus research	22.2	https://www.springernature.com/gp/researchers/campaigns/coronavirus
UKRI Coronavirus Explained	20.8	https://coronavirusexplained.ukri.org/en/
LitCovid NCBI Journal Articles	11.1	https://www.ncbi.nlm.nih.gov/research/coronavirus/
Science—Coronavirus research	8.3	https://www.sciencemag.org/collections/coronavirus?IntCmp=coronavirussiderail-128
Unique. Rarechromo.org	6.9	https://www.rarechromo.org/covid19update
NHS Education for Scotland	5.6	https://learn.nes.nhs.scot/27993/coronavirus-covid-19
World Health Organisation	4.2	https://www.who.int/
RCSB Protein Data Bank	4.2	http://www.rcsb.org/
British Soc. for Genetic Medicine	2.8	https://www.bsgm.org.uk/
National University of Singapore	2.8	https://sph.nus.edu.sg/covid-19/research/

**FIGURE 2:**
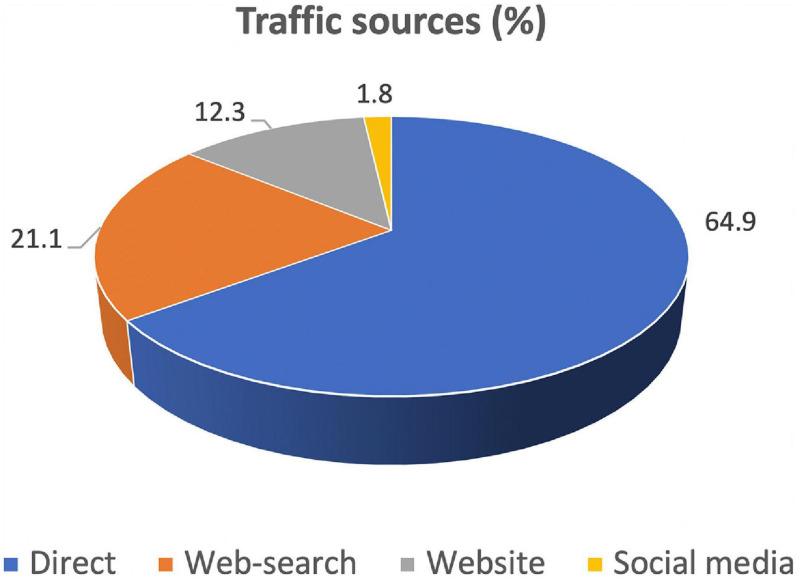
Proportions of visits to ScotGEN’s COVID-19 web pages attributable to different website traffic sources.

Most visitors reached the ScotGEN pages directly via URL (64.9%) but just over a third reached the pages in other ways: 21.1% via web searches (for terms including “covid learning resources,” of which 79.3% were performed using Google, 18.9% by Bing, 0.6% by DuckDuckGo, 0.6% by Ecosia and 0.6% by Yahoo), 12.3% via website referral and 1.8% from social media sources, principally Facebook and Twitter (Figure 2).

The exit links that were most frequently used, in order of decreasing frequency, were: Nature Journal coronavirus research (22.2%), UKRI Coronavirus Explained (20.8%), PubMed’s LitCovid COVID-19 Articles (11.1%), Science Journal coronavirus research (8.3%), and the Unique/rarechromo.org (6.9%) website that provides guidance for individuals affected by rare genetic disorders and their families.

### Testimonials

Feedback received with regard to the new pages has been highly positive, including, for example, the following comments from genetics consultants: “Looks very good to me”; “Very useful and what a good idea to bring all the resources together like this”; “Easy to navigate and what I have read is very readable and clear”; “Excellent”; and “Fabulous.” Those comments were received after the webpages were presented in detail to many clinical genetic professionals, highly experienced in communicating with patients and their relatives.

## Discussion

Two new web-pages have been created on the ScotGEN website that have already been used by a large number of individuals from over 50 countries, worldwide. It is envisaged that providing a range of links to useful COVID-19 resources, in particular those relating to genetics, from a single hub, together with a brief summary of each website’s content, will make it easier and quicker for visitors to access a range of sources of information relevant to them. This would appear to be the case already, from the feedback received, by the rapidly increasing number of returning visitors and their wide geographical distribution.

The web pages’ links are continually checked to ensure the absence of “404” or “page not found” errors and where necessary, URLs are updated. New links have continued to be added since the original launch of the web pages on 24th April 2020, providing easy access for professionals and the public to additional information provided from sources located around the world. The authors would, however, welcome emailed suggestions for additional links to high-quality freely accessible online COVID-19 information sources.

Further development is planned, including the provision of additional resources and the further growth of the web pages’ content.

The authors hope that readers will inform other individuals, including colleagues and members of the public, of the pages’ existence, in order to maximise the number of people who can benefit from this free information hub.

## Data Availability Statement

The latest version of the information hub presented in the article, together witht its full content and all of its links to online resources, are freely available online at www.ScotGEN.org.uk. Please direct any further inquiries to the corresponding author.

## Author Contributions

AT and ET conceived, designed and created the COVID-19 web pages, analysed the data, created the figures and tables, and wrote the manuscript. JB, RC, ZM, and MP assisted with the page content selection and the incorporation and hosting of the web pages on the ScotGEN website and the acquisition of the necessary ScotGEN funding. All authors read and approved the final manuscript.

## Conflict of Interest

The authors declare that the research was conducted in the absence of any commercial or financial relationships that could be construed as a potential conflict of interest.
